# Loss of the Promyelocytic Leukemia Protein in Gastric Cancer: Implications for IP-10 Expression and Tumor-Infiltrating Lymphocytes

**DOI:** 10.1371/journal.pone.0026264

**Published:** 2011-10-12

**Authors:** Hee Ja Kim, Dong Eun Song, Seul Ye Lim, Sung-Hee Lee, Jihee Lee Kang, Sun Jung Lee, Etty N. Benveniste, Youn-Hee Choi

**Affiliations:** 1 Department of Physiology, School of Medicine, Ewha Womans University, Seoul, South Korea; 2 Tissue Injury Defense Research Center, School of Medicine, Ewha Womans University, Seoul, South Korea; 3 Department of Pathology, University of Ulsan College of Medicine, Asan Medical Center, Seoul, South Korea; 4 Department of Pathology, Harvard Medical School, Boston, Massachusetts, United States of America; 5 Department of Cell Biology, University of Alabama at Birmingham, Birmingham, Alabama, United States of America; Veterans Affairs Medical Center (111D), United States of America

## Abstract

Gastric cancer is one of the most common causes of cancer-related mortality worldwide. Expression of the tumor suppressor, promyelocytic leukemia (PML) protein, is reduced or abolished in gastric carcinomas, in association with an increased level of lymphatic invasion, development of higher pTNM staging, and unfavorable prognosis. Herein, we investigated the relationship between the extent of tumor-infiltrating lymphocytes and the status of PML protein expression in advanced gastric carcinoma. We observed higher numbers of infiltrating T-cells in gastric carcinoma tissues in which PML expression was reduced or abolished, compared to tissues positive for PML. The extent of T-cell migration toward culture supernatants obtained from interferon-gamma (IFN-γ-stimulated gastric carcinoma cell lines was additionally affected by expression of PML *in vitro*. Interferon-gamma-inducible protein 10 (IP-10/CXCL10) expression was increased in gastric carcinoma tissues displaying reduced PML levels. Moreover, both *Pml* knockout and knockdown cells displayed enhanced IP-10 mRNA and protein expression in the presence of IFN-γ. PML knockdown increased IFN-γ-mediated Signal Transducer and Activator of Transcription-1 (STAT-1) binding to the IP-10 promoter, resulting in elevated transcription of the IP-10 gene. Conversely, PML IV protein expression suppressed IP-10 promoter activation. Based on these results, we propose that loss of PML protein expression in gastric cancer cells contributes to increased IP-10 transcription *via* enhancement of STAT-1 activity, which, in turn, promotes lymphocyte trafficking within tumor regions.

## Introduction

Gastric cancer is one of the most frequent causes of cancer-related deaths worldwide. Recent advances in genetics have facilitated the detection of events occurring during the course of gastric carcinogenesis, including activation of oncogenes, silencing of tumor suppressor genes, and mutation of genes involved in DNA repair [1]. Among these genetic events, it is known that tumor suppressor promyelocytic leukemia (PML) protein expression is reduced or abolished in gastric cancer, and that this is associated with more extensive lymphatic invasion, higher pTNM staging, and unfavorable prognosis, suggesting that PML loss is linked to carcinogenesis and gastric carcinoma progression [2]. Earlier studies implicated *Pml* dysfunction in progression of a variety of cancers, and reduced or abolished expression of PML has been reported in prostate, breast, CNS, colon, lung, and gastric cancers [2], [3].

The *Pml* gene was originally identified by fusion with the retinoic acid receptor involved in the t(15;17) chromosomal translocation associated with acute promyelocytic leukemia (APL) [4], [5], [6], [7], [8]. PML is a tumor suppressor protein that regulates cell cycle progression, gene transcription, transformation suppression, and apoptosis. *Pml* knockout mice are considerably more sensitive to tumor formation after exposure to carcinogens than are wild-type controls, and *Pml*-deficient cells are less likely to undergo apoptosis after application of particular types of cellular stress [9]. In addition to reports focusing on the tumor suppressor role played by PML, several studies have confirmed that the protein acts as a transcriptional regulator, *via* association with the co-activator CREB-binding protein; co-repressors including HDAC, N-CoR, and mSin3A; and the transcription factors Nur77, AP-1, myc, p53, and STAT-1α [10], [11], [12], [13], [14], [15], [16], [17].

Progressive mutation of and genetic alterations in several tumor suppressor genes promote tumor development and progression [18]. In addition to genetic alterations within tumor cells, both inflammatory cells and mediators contribute to formation of particular tumor microenvironments [19], [20]. Tumor-associated fibroblasts (TAFs) and macrophages (TAMs) are also involved in modulation of the tumor microenvironment *via* production of cytokines, chemokines, interferons, and other biologically active factors [21], [22]. Cross-talk among tumor cells, TAFs, TAMs, and lymphocytes, is significant in tumor development and progression, and contributes to establishment of tumor microenvironments enriched in expression of a variety of biologically active factors [23], [24], [25], [26], [27].

TAMs are found to correlate with angiogenesis and an unfavorable prognosis in several types of cancer, including gastric cancer [22], [28]. Mast cell produces many angiogenic factors and a variety of cytokines, and its density has been reported to highly correlate with the extent of tumor neoangiogenesis and poor prognosis [29], [30]. CD4^+^ and CD8^+^ lymphocytes are also found in a variety of solid cancer tissues. Until now, the precise molecular mechanisms by which the type and the number of tumor infiltrating cells are regulated are largely unknown. There are several controversial reports with regard to the number and function of tumor-infiltrating lymphocytes. Especially, CD8^+^ T lymphocytes, while their function is known to eliminate nascent transformed cells and contribute to immunosurveillance [31], it is reported that CD8^+^ tumor-infiltrating T cells are defective in effector phase function upon contact with tumor cells [32], and their infiltration is linked to unfavorable prognosis in certain types of cancer such as nonsmall cell lung cancer and nasopharyngeal carcinoma [33], [34].

Chemokines secreted by stromal cells or tumor cells influence tumor cell migration, invasion, proliferation, angiogenesis and immune cell infiltration in the tumor mass [35]. IFN-γ-inducible protein-10 (IP-10, CXCL10) is one of the CXC chemokines which plays multiple roles in inflammatory diseases and cancer [36], [37]. Since IP-10 preferentially promotes Th1 immune responses by robustly attracting NK and T cells, it is suggested as one of possible mechanisms of T cell infiltration to tumors.

Whether loss of tumor suppressor genes in tumor cells induces recruitment of lymphocytes and modulation of the functions of such cells within tumor microenvironments is currently unclear. In the present study, we examined PML function with respect to recruitment of lymphocytes and modulation of the expression of tumor-derived factors. Expression of PML was inversely correlated with the extent of infiltration of CD8^+^ T-cells into gastric carcinomas. PML loss was further associated with enhanced expression of IP-10, one of the lymphocyte-attracting CXC chemokines, in gastric carcinoma cells and tissues. PML knockdown promoted IFN-γ-mediated STAT-1 binding to the IP-10 promoter, resulting in increased transcription of the IP-10 gene. Our data support the notion that PML is involved in recruitment of lymphocytes into tumors, *via* modulation of the expression levels of tumor-derived factors including IP-10, providing further evidence that the loss of tumor suppressor genes in tumor cells actively participates in the establishment of tumor microenvironments.

## Results

### Loss of PML protein is associated with increased infiltration of CD8^+^ T-cells into advanced gastric carcinoma tissue

**Figure 1:**
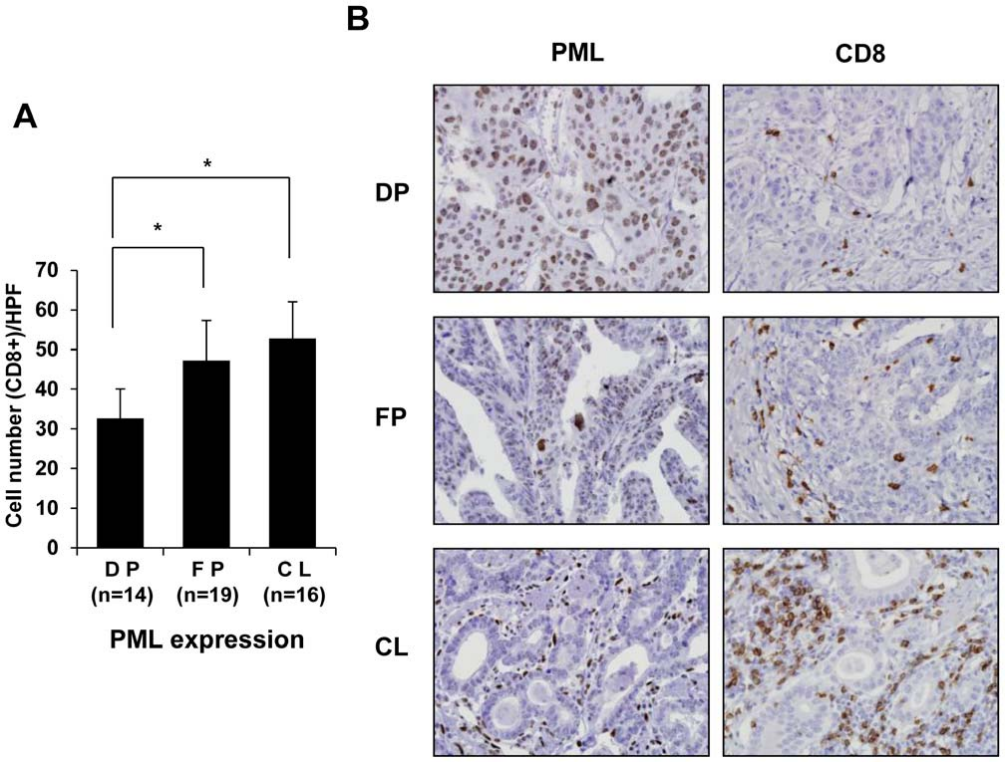
Promyelocytic Leukemia (PML) Protein Expression is Inversely Correlated with the Extent of Infiltration of CD8^+^ T-cells in Advanced Gastric Carcinoma Tissues. (A) Immunohistochemical staining for PML protein and CD8 was performed for 49 cases of stage IV advanced gastric carcinomas. Immunopositivity for PML protein was categorized as diffuse positivity (DP; nuclear immunoreactivity in ≥50% of tumor cells, n = 14), focal positivity (FP; in ≥10% but <50%, n = 19), or complete loss (CL; in <10%, n = 16). The number of CD8 immunopositive cells in one high power field (HPF, x40) of 0.25 mm^2^ for five randomly selected tumor infiltrative borders were counted for each specimen, followed by calculation of the mean and SD. (B) Representative images showing PML protein expression and CD8^+^ T-cell infiltration in advanced gastric carcinoma tissues. Bars, SD. *P<0.05.

Immunohistochemical staining for PML and CD8 was performed to explore whether the extent of lymphocyte infiltration was associated with PML expression status in advanced gastric cancer. CD8-immunopositive cells were enumerated as described in Materials and Methods. In total, 35 samples (71.4%) showed partial-to-complete loss of PML in the tumor cell nuclei of stage IV advanced gastric carcinomas. Samples from 14 advanced gastric carcinoma patients were diffusely positive for PML, and contained an average of 32.7 CD8^+^ T-cells per field (Figure 1A). We identified 19 instances of advanced gastric carcinoma exhibiting focal positivity for PML, with an average of 47.2 CD8^+^ T-cells per field. Moreover, complete loss of PML was observed in 16 advanced gastric carcinoma patients; the samples contained an average number of 52.8 CD8^+^ T-cells per field. Samples displaying focal positivity or complete loss for PML showed a more marked increase in CD8^+^ T-cell number than did specimens exhibiting diffuse positivity for PML. Representative images are shown in Figure 1B. Although PML protein expression was reduced or abolished in tumor cells, all of normal gastric mucosal glands, stromal tissues, and lymphoid cells, exhibited diffuse moderate-to-strong nuclear immunopositivity for PML.

### PML influences T-cell infiltration/migration *in vitro*

**Figure 2:**
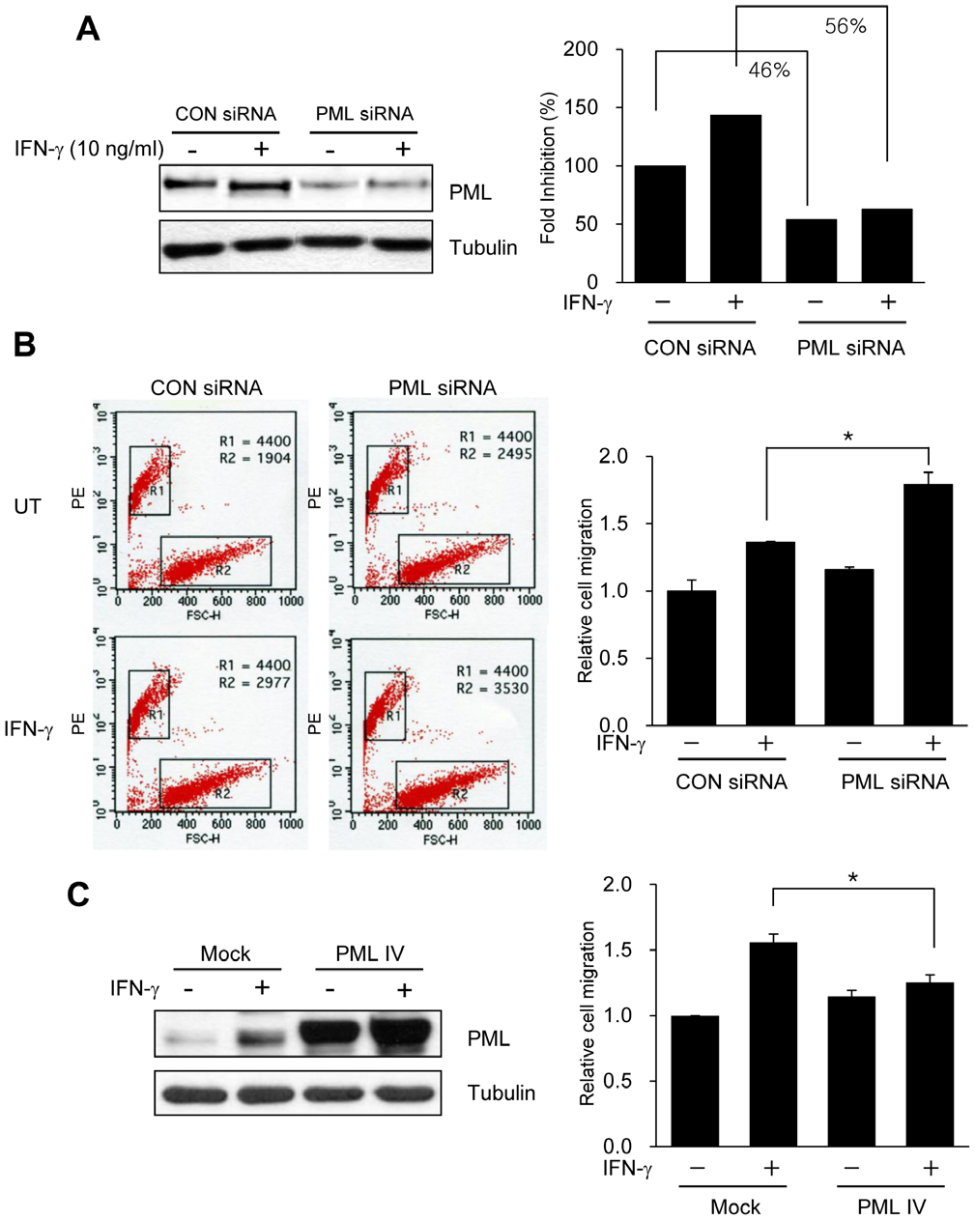
Reduced PML Protein Expression by siRNA Increases T-cell Infiltration/Migration. (A) SNU-638 gastric cancer cells were transiently transfected with *Pml* siRNA or control siRNA. Two days after transfection, cells were treated with or without interferon-gamma (IFN-γ (10 ng/ml) for 8 h and lysed and analyzed by immunoblotting. Effective siRNA-mediated suppression of PML protein expression was verified for each assay by immunoblotting. (B) Jurkat cells and culture supernatants from SNU-638 cells which were transiently transfected with either control siRNA or *Pml* siRNA were analyzed by a transwell migration assay. After transfection, SNU-638 cells were incubated with IFN-γ (10 ng/ml) for 8 h or left untreated, and the collected supernatants were placed in the lower chambers. The upper chambers received 5×10^5^ Jurkat cells in a volume of 100 µl and the transwell migration units were incubated at 37°C for 2 h. Migration of Jurkat cells from the upper to lower chamber was analyzed by examining cells harvested from the lower chambers by fluorescent count-bead mediated counting on a flow cytometer. Relative cell migration was determined by the number of migrated cells normalized to the number of cells migrating to supernatants from SNU-638 cells transiently transfected with control siRNA without IFN-γ, and the value from parental cells was arbitrarily set at 1. (C) SNU-638 gastric cancer cells were transiently transfected with either empty vector (Mock) or PML IV expression vector (PML IV). Day after transfection, cells were treated with or without IFN-γ (10 ng/ml) for 8 h and culture supernatants were obtained and subjected to transwell migration assay as described in B. PML protein expression was verified for each assay by immunoblotting. Data shown are representative of at least three experiments. Bars, SD. *P<0.05.

In view of the loss of PML protein expression and the increase in infiltration of CD8^+^ T-lymphocytes into advanced gastric carcinoma tissues, we hypothesized that PML contributed to T-cell infiltration/migration in gastric cancer. To explore whether the PML level in gastric cancer cells influenced T-cell migration, conditioned media from SNU-638 cells transiently transfected with either *Pml* siRNA or PML IV expression vector, then treated with IFN-γ was utilized in transwell migration assays. SNU-638 gastric cancer cells expressed high levels of PML protein, consistent with previous findings [2], whereas SNU-638 cells transfected with *Pml* siRNA displayed reduced levels of endogenous PML expression (∼46–56%), as confirmed by immunoblotting (Figure 2A). IFN-γ induced a modest increase in PML protein expression, in agreement with previous results [38]. Migration of Jurkat T-cells toward conditioned medium from *Pml* siRNA-transfected SNU-638 cells was significantly enhanced, compared to that of control siRNA-transfected cells, after treatment with IFN-γ (Figure 2B). Upon transfection of SNU-638 with PML IV expression vector, migration of Jurkat T-cells toward conditioned medium from PML IV overexpressed cells was reduced compared to that of empty vector (Figure 2C). These results suggest that PML regulates the levels of secretory molecules produced by and released from IFN-γ-stimulated gastric cancer cells, and also influences the migration of T- lymphocytes.

### Loss of PML enhances IFN-γ-induced IP-10 expression

**Figure 3:**
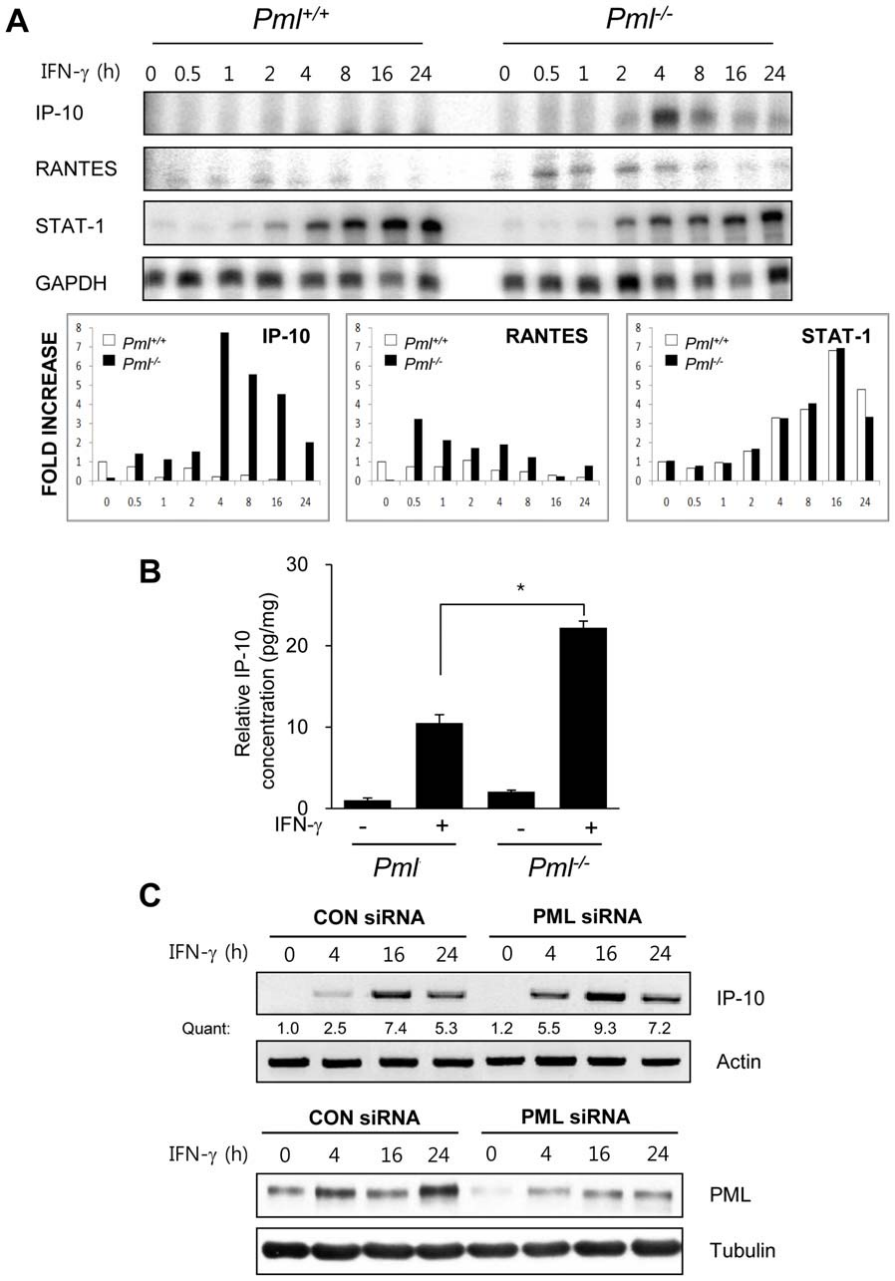
Expression Levels of IFN-γ-inducible protein-10 (IP-10) are Enhanced in *Pml* ^−/−^ **MEFs compared to** ***Pml^+/+^*** **wild-type cells.** (A) RNA from *Pml^+/+^ and Pml* ^−/−^ MEFs treated with IFN-γ (10 ng/ml) for 0-24 h was subjected to ribonuclease protection assay (RPA). Quantification of IP-10, RANTES and STAT-1 mRNA (FOLD INCREASE) was calculated by dividing the densitometric value of each lane by the corresponding GAPDH value. (B) *Pml^+/+^* and *Pml* ^−/−^ MEFs cells were incubated in the absence or presence of IFN-γ (10 ng/ml) for 12 h. IP-10 protein from the culture supernatants were analyzed by ELISA and normalized by the cell protein concentration. The relative concentration was calculated as the normalized amount divided by the normalized amount of untreated *Pml* ^−/−^ MEFs cells. (C) NIH3T3 fibroblasts were transiently transfected with either *Pml* siRNA or control siRNA. Two days after transfection, cells were treated with or without IFN-γ for 0-24 h, and analyzed by immunoblotting for detection of PML protein expression and RT-PCR for IP-10 mRNA. Quantification (Quant) of IP-10 mRNA was calculated by dividing the densitometric value of each lane by the corresponding actin mRNA value. Data shown are representative of at least three experiments. Bars, SD. *P<0.01.

Chemokines, a family of chemotactic cytokines, promote migration of circulating leukocytes/lymphocytes to sites of inflammation and injury. To explore whether chemokine levels were affected by *Pml* genetic status, chemokine gene expression was examined using ribonuclease protection assay (RPA). *Pml* ^+/+^ and *Pml* ^−/−^ mouse embryonic fibroblast (MEF) cells were incubated in the absence or presence of IFN-γ for 0-24 h, total mRNA was collected at various timepoints, and subjected to RPA. In *Pml* ^−/−^ MEFs, IP-10 mRNA levels increased 1.5-fold at 2 h, and became markedly elevated (8-fold) by 4 h (Figure 3A). RANTES mRNA levels were increased 3.2-fold at 0.5 h, and remained elevated, but to a lower extent (1.3-fold), 8 h post-IFN-γ treatment in *Pml* ^−/−^ MEFs. Other chemokine genes, including MIP-1, MIP-2, and MCP-1, were not detectably expressed in *Pml* ^+/+^ or *Pml* ^−/−^ MEFs (data not shown). STAT-1 expression was increased after exposure to IFN-γ in both cell types, but no difference was evident when IFN-γ levels were compared between *Pml* ^+/+^ and *Pml* ^−/−^ cells. As transcription of the IP-10 gene was substantially elevated in IFN-γ-stimulated *Pml* ^−/−^ MEFs, we measured relevant protein levels in culture supernatants of *Pml* ^+/+^ and *Pml* ^−/−^ MEFs, using an enzyme-linked immunosorbent assay (ELISA). Consistent with the RPA results, higher levels of IP-10 protein were evident in conditioned medium from IFN-γ-treated *Pml* ^−/−^ MEFs (Figure 3B). Also, IP-10 mRNA expression in *Pml* siRNA-transfected NIH3T3 cells was enhanced, compared to that of control siRNA-transfected cells, following IFN-γ treatment (Figure 3C). The reduction in expression of PML protein in *Pml* siRNA-transfected NIH3T3 cells was confirmed by immunoblotting.

### Reduced PML expression is inversely correlated with IP-10 protein levels in gastric cancer tissue and SNU-638 cells

**Figure 4:**
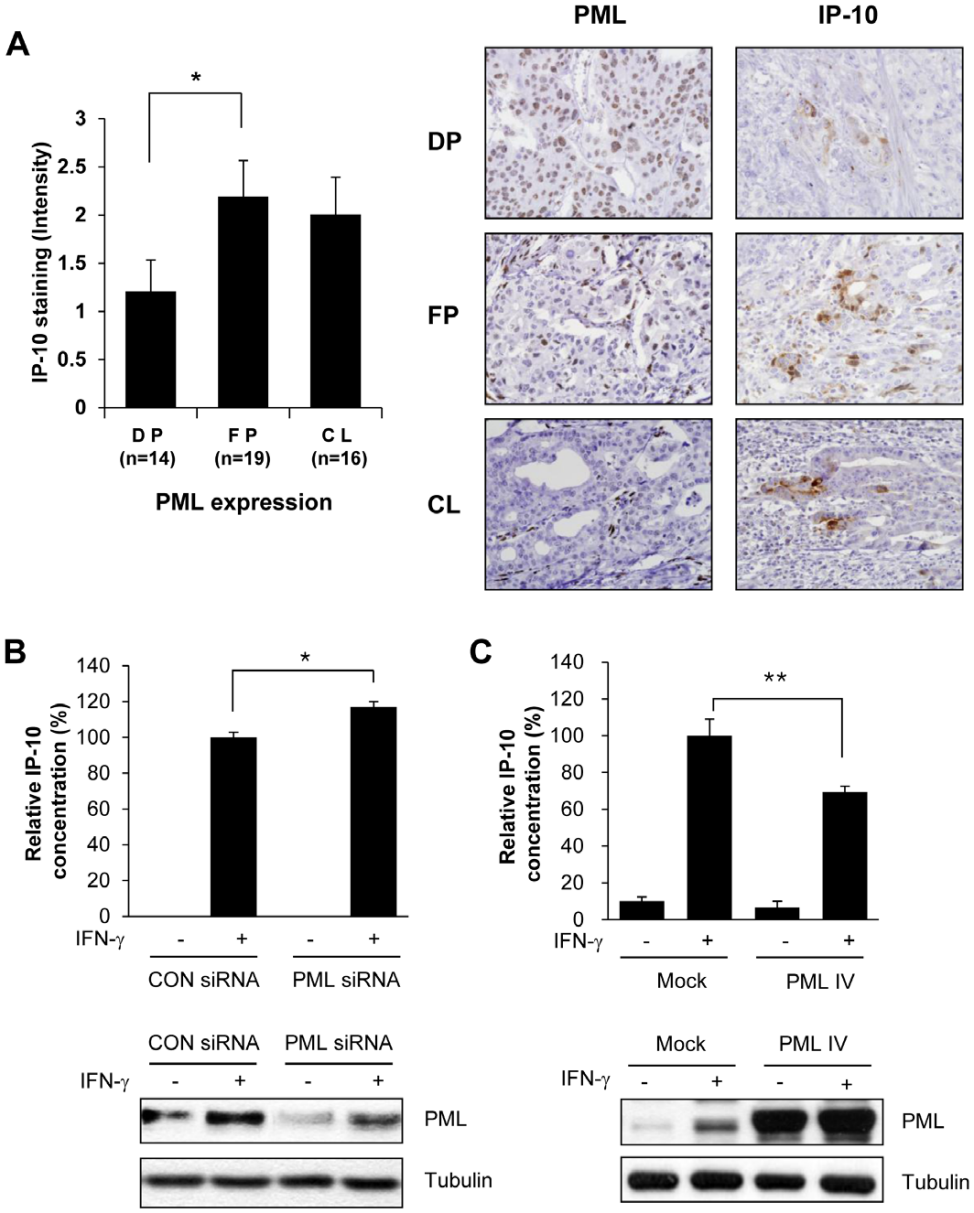
PML Protein Expression is Inversely Correlated with the Level of IP-10. (A) Immunohistochemical staining for PML and IP-10 protein expression was performed for 49 cases of stage IV advanced gastric carcinomas. Immunopositivity for PML protein was categorized as diffuse positivity (DP; nuclear immunoreactivity in ≥50% of tumor cells), focal positivity (FP; in ≥10% but <50%), or complete loss (CL; in <10%). For quantitative analysis of immunoreactivity of IP-10, positively stained areas were measured using an ImageJ software program as described in Materials and Methods. Representative images show PML and IP-10 protein expression in advanced gastric carcinoma tissues (right). (B) SNU-638 cells were transiently transfected with *Pml* siRNA or control siRNA. Two days after transfection, cells were incubated in the absence or presence of IFN-γ (10 ng/ml) for 8 h. IP-10 protein from the culture supernatants was analyzed by ELISA and normalized by the cell protein concentration. The relative concentration was calculated as the normalized amount divided by the normalized amount of SNU-638 cells which were transiently transfected with control siRNA in the presence of IFN-γ, and the concentration from parental cells was arbitrarily set at 100%. (C) SNU-638 gastric cancer cells were transiently transfected with either empty vector or PML IV expression vector. Day after transfection, cells were treated with or without IFN-γ (10 ng/ml) for 8 h and culture supernatants were obtained and subjected to ELISA as described in B. PML protein expression was verified for each assay by immunoblotting. Data shown are representative of at least three experiments. Bars, SD. *P<0.05, **P<0.01.

Next, we examined the relationship between IP-10 and PML protein expression in advanced gastric carcinoma tissue. Cancer tissues were immunohistochemically stained for PML and IP-10, and the intensity of IP-10 immunopositivity was measured as described in Materials and Methods. The average intensity of IP-10 was 1.2 in 14 samples showing diffuse positivity (DP) for PML, 2.2 in 19 instances of focal positivity (FP), and 2.0 in 16 samples in which there was complete loss (CL) of PML expression (Figure 4A, left panel). Significant increases in IP-10 expression were observed in samples showing focal positivity of PML (*P* = 0.034), compared to those showing diffuse positive PML expression. An increase in IP-10 expression was observed when complete loss of PML was evident, compared with what was noted when PML was diffusely expressed; however, this difference did not reach statistical significance. Representative examples of variations in PML protein status that correlated with different IP-10 expression levels in tumor cells from advanced gastric carcinomas are shown (Figure 4A, right). To further examine the relationship between IP-10 and PML protein expression in gastric carcinoma cell lines, IP-10 protein levels were measured in culture supernatants from SNU-638 cells transiently transfected with either *Pml* siRNA or PML IV exression vector. Cells were grown in the absence or presence of IFN-γ for 8 h, supernatants collected, and IP-10 levels quantified therein (by ELISA). IFN-γ-induced IP-10 secretion was significantly enhanced in SNU-638-*Pml* siRNA-transfected cells, compared with that of SNU-638 cells transfected with control siRNA (Figure 4B). Transfection of SNU-638 with PML IV expression vector suppressed IFN-γ-induced IP-10 secretion (Fig 4C). The results clearly show that secretion of IP-10 from gastric cancer cells is increased when PML expression is reduced.

### IP-10 neutralization decreases migration of T-cells

**Figure 5:**
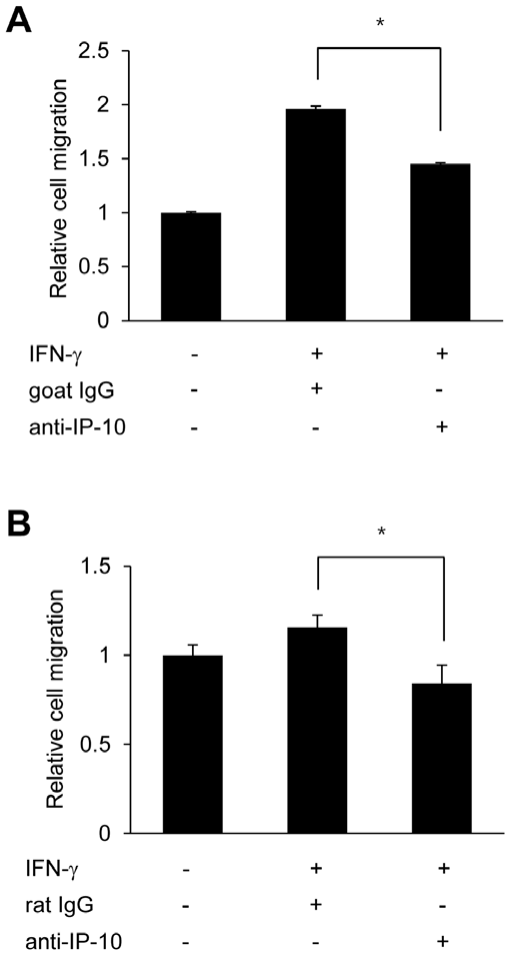
IP-10 Neutralization Inhibits the Migration of T Cells. (A) SNU-638-*Pml* siRNA cells were grown in the absence or presence of IFN-γ (10 ng/ml) for 8 h, and supernatant was collected and pretreated with IP-10 neutralizing antibodies (5 µg/ml) or IgG (5 µg/ml) for 1 h prior to initiation of the transwell migration assay. The number of migrating Jurkat cells obtained from the lower chamber was analyzed by fluorescent count-bead mediated counting on a flow cytometer. (B) Transwell migration of EL-4 cells to culture supernatants from NIH3T3 cells transiently transfected with *Pml* siRNA in the presence of neutralizing IP-10 antibody or IgG control. Relative cell migration was determined by the number of the migrated cells divided by the number of cells migrating to supernatants without IFN-γ, and the value from parental cells was arbitrarily set at 1. Data shown are representative of at least three experiments. Bars, SD. *P<0.05.

As SNU-638-*Pml* siRNA-containing cells expressing reduced levels of PML protein showed enhancement of T-cell recruitment and IP-10 secretion in response to IFN-γ (Figures 2 and 4), we next examined whether an increase in IP-10 levels facilitated T-cell migration toward conditioned medium from SNU-638 gastric cancer cells stimulated by IFN-γ. SNU-638-*Pml* siRNA cells were grown in the absence or presence of IFN-γ for 8 h, and the collected supernatants were pretreated with an anti-IP-10 neutralizing antibody or control non-specific IgG for 1 h, prior to commencement of the transwell assay. Neutralizing antibody or IgG-containing supernatants were placed in the lower chambers, and migration of Jurkat cells from upper to lower chambers was analyzed by examining cells harvested from the lower chambers. Inclusion of IP-10-neutralizing antibodies inhibited Jurkat T-cell migration toward conditioned medium from SNU-638-*Pml* siRNA cells (Figure 5A). These findings are consistent with data obtained from NIH3T3 cells; an IP-10-neutralizing antibody inhibited EL-4 T-cell migration toward conditioned medium from NIH3T3-*Pml* siRNA cells (Figure 5B).

### PML knockdown enhances IP-10 promoter activation

**Figure 6:**
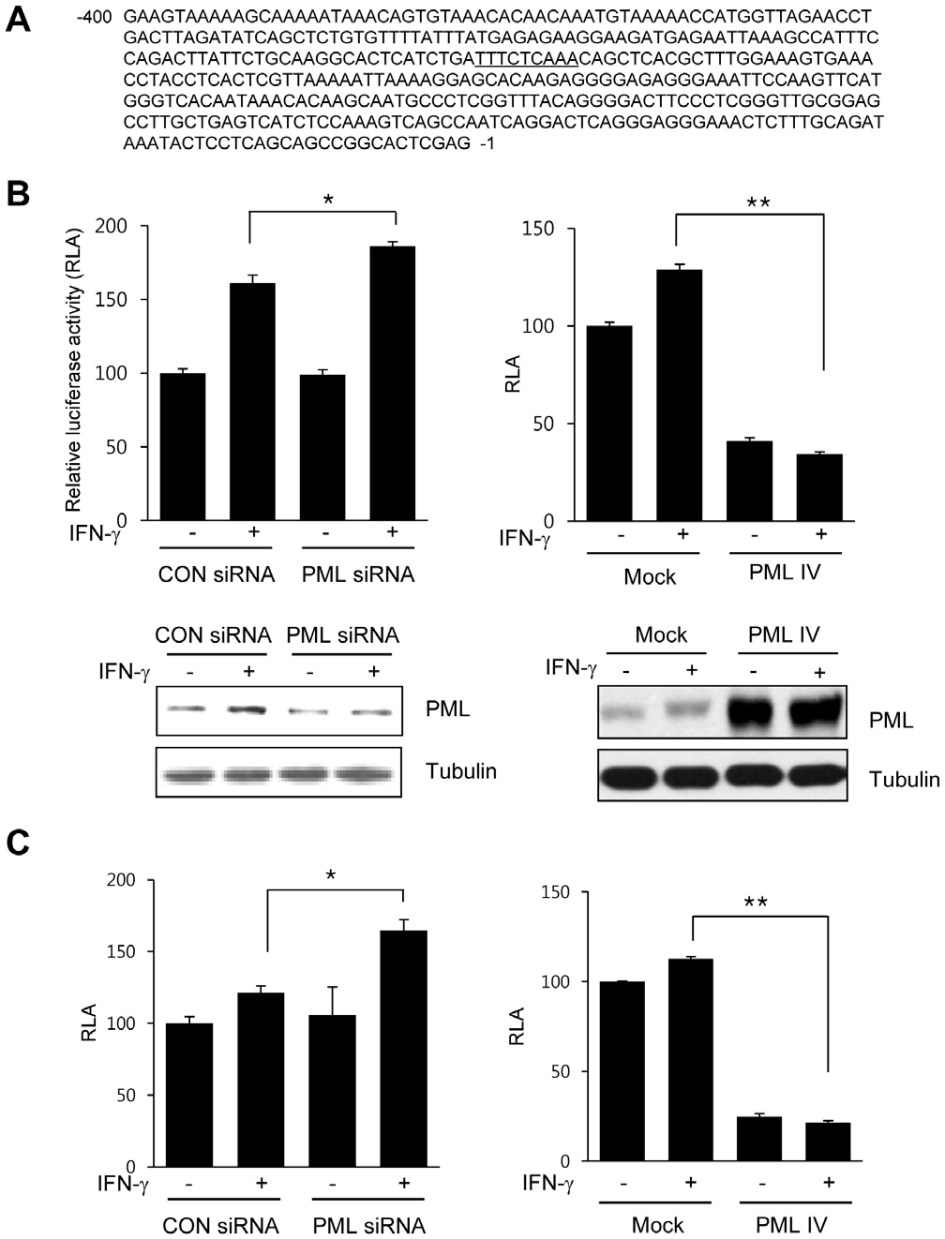
PML Expression Represses IFN-γ-inducible IP-10 Promoter Activity. (A) The murine IP-10 promoter contains a putative GAS-like element (underlined). (B) NIH3T3 cells were co-transfected with the IP-10-luc reporter containing a GAS-like element and/or *Pml* siRNA, or the PML IV protein expression vector. Cells were either untreated or treated with murine IFN-γ (10 ng/ml) for 24 h, and then luciferase activity was determined. The pCMV-β-galactosidase vector was included to normalize transfection efficiency. Luciferase activity is normalized to the activity in the absence of IFN-γ and the activity from parental cells was arbitrarily set at 100% (RLA; relative luciferase activity). Increased or reduced PML protein expression in cell lysates transfected with either PML IV expression vector or *Pml* siRNA was verified by immunoblotting, respectively. (C) SNU-638 gastric cancer cells were co-transfected with the IP-10-luc reporter containing a GAS-like element and/or *Pml* siRNA, or the PML IV protein expression vector and analyzed as described in B. Values are the mean ± SD of three separate experiments. Bars, SD. *P<0.05, **P<0.001.

As IP-10 expression was increased in PML knockdown cells, we further explored whether PML protein expression affected IP-10 promoter activity. The murine IP-10 promoter contains a putative GAS-like element lying between nts -246 and -238 (TTN_5_AA) (Figure 6A). We previously reported that PML negatively regulates the transcriptional activity of STAT-1[17]. To ascertain whether PML affected IFN-γ-induced IP-10 gene expression *via* a mechanism involving STAT-1, we isolated and generated a reporter construct commencing at position -330 of the murine IP-10 promoter containing the putative GAS-like element, as described in Materials and Methods. The IP-10-*luc* reporter containing the GAS-like element was transiently transfected into NIH3T3 cells incubated with or without IFN-γ for 24 h, and subsequently assayed for luciferase activity. IFN-γ-induced IP-10 promoter activity was significantly enhanced in NIH3T3 cells transfected with *Pml* siRNA, compared to cells receiving control siRNA (Figure 6B). Upon transfection of NIH3T3 cells with the PML IV expression vector, IFN-γ-induced IP-10 promoter activation was significantly repressed. Downregulation of PML expression using *Pml* siRNA and overexpression of PML by PML IV expression vector were verified by immunoblotting. To determine whether the inhibitory effect of PML on IFN-γ-induced IP-10 promoter activity is occurred in gastric cancer cells lines, we performed similar experiments with SNU-638 gastric cancer cells. The patterns of increase and decrease in the luciferase activity in SNU-638 were similar to that of NIH3T3 cells (Figure 6C). The level of PML protein expression in SNU-638 cells transfected with either *Pml* siRNA or PML IV expression vector was verified by immunoblotting (data not shown).

### PML knockdown increases IFN-γ-induced STAT-1 DNA binding to the IP-10 promoter

**Figure 7:**
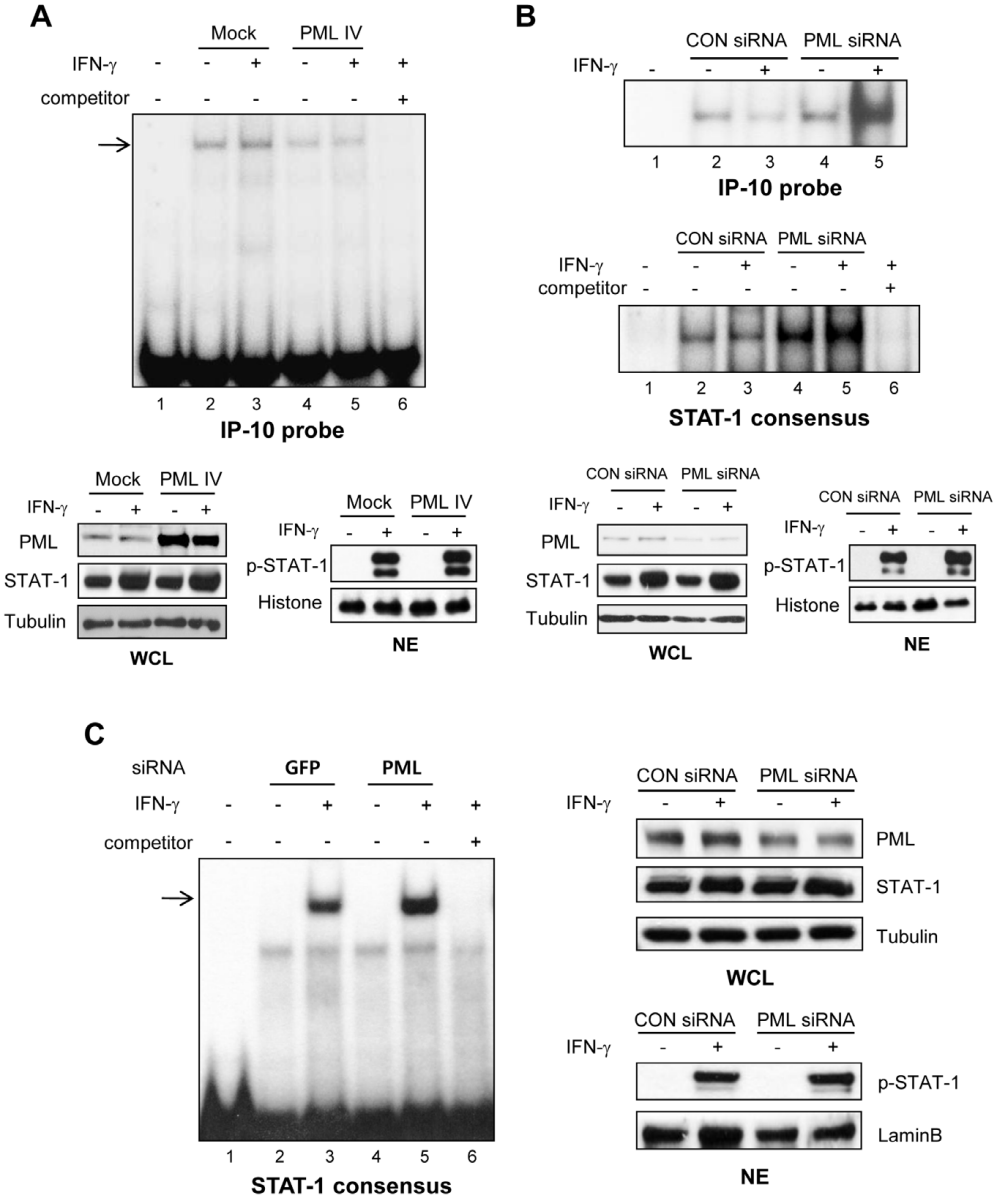
PML Expression Inhibits DNA Binding Activity of STAT-1 to the IP-10 Promoter. (A) Nuclear extracts (NE) from NIH3T3 cells transiently transfected with the PML IV protein expression vector and treated with IFN-γ (10 ng/ml) for 30 min were incubated in the presence of a radiolabeled DNA probe containing the GAS-like element of the IP-10 promoter, and subjected to electrophoretic mobility shift assay (EMSA). Shifted probe-protein complexes are indicated by the arrow. Increased PML protein expression in whole-cell lysates (WCL) transfected with PML IV expression vector was verified by immunoblotting. The amount of histone in NE is shown as a control. (B) NEs from NIH3T3 cells transiently transfected with either *Pml* siRNA or control siRNA and treated with IFN-γ (10 ng/ml) for 30 min were incubated in the presence of radiolabeled DNA probes containing the GAS-like element of the IP-10 promoter or a STAT-1 consensus sequence, and then subjected to EMSA. Binding specificity was tested by adding increasing concentrations of cold probe (competitor). Effective siRNA-mediated suppression of PML protein expression was verified for each assay by immunoblotting. (C) SNU-638 gastric cancer cells were transiently transfected with either *Pml* siRNA or control siRNA and treated with IFN-γ (10 ng/ml) for 30 min. NEs were obtained, incubated in the presence of radiolabeled DNA probes containing a STAT-1 consensus sequence, and then analyzed for EMSA as described in B. The levels of PML and STAT-1 protein expression in WCL and the extent of STAT-1 tyrosine phosphorylation in NE were verified by immunoblotting. Data shown are representative of at least three experiments.

The effect of PML on IFN-γ-induced STAT-1 DNA binding to the IP-10 promoter was further examined. PML protein overexpression in NIH3T3 cells, after transient transfection with the PML IV expression vector, partially blocked IFN-γ-induced STAT-1 DNA binding to the IP-10 promoter, including the sequence at -246 to -238 (Figure 7A, compare lanes 3 and 5). Competition using a 100-molar excess unlabeled oligonucleotide encoding the IP-10 promoter abrogated complex formation (lane 6). The levels of PML and STAT-1 protein expression in whole-cell lysates (WCL) and the extent of STAT-1 tyrosine phosphorylation in nuclear extracts (NE) were verified by immunoblotting. Utilizing NE from NIH3T3-*Pml* siRNA cells obtained after 0.5 h of IFN-γ treatment, strong binding to the murine IP-10 promoter was observed (Figure 7B, top panel, lane 5), compared to binding seen in NIH3T3-control siRNA cells (lane 3). The same NEs were exposed to the STAT-1 consensus oligonucleotide, and STAT-1 DNA binding was enhanced in NE from NIH3T3-*Pml* siRNA cells (Figure 7B, middle panel, compare lanes 3 and 5). Using SNU-638 gastric cancer cells, we also found that STAT-1 DNA binding of *Pml* siRNA-transfected SNU-638 cells was enhanced compared to that of control siRNA-transfected cells, after treatment with IFN-γ (Figure 7C, compare lanes 3 and 5). These data indicate that PML partially inhibits STAT-1 binding to the IP-10 promoter, and that this effect is correlated with increased IFN-γ-induced IP-10 transcription in the absence of PML.

## Discussion

Histopathologic studies have shown that the inflammatory microenvironment of tumors is characterized by the presence of mononuclear cell infiltrates, both in the supporting stroma and tumor regions. However, the precise mechanisms by which mononuclear cells infiltrate into and are sustained within cancer tissues are currently not fully understood. In the present study, we offer evidence that loss of expression of the tumor suppressor protein, PML, contributes to enhancement of lymphocyte infiltration into gastric cancer tissue, *via* regulation of IP-10 expression.

We examined the function of PML with respect to lymphocyte recruitment using human gastric cancer tissue samples, *Pml* ^+/+^ and *Pml* ^−/−^ MEFs, and PML knockdown in both NIH3T3 and SNU-638 cells. Data obtained from both human tissue tests and the transwell migration assays showed that loss of PML promotes lymphocyte trafficking; thus, we sought mechanisms that might possibly explain these observations. We show that an increase in IP-10 levels in cancer cells expressing low levels of PML contributes to lymphocyte recruitment. Additionally, IP-10 neutralizing antibody inhibited T-cell migration toward conditioned medium from NIH3T3 fibroblasts and SNU-638 gastric cancer cells *in vitro*, consistent with the results of several prior *in vivo* studies, which showed that neutralization of IP-10 suppressed T-cell recruitment [39], [40]. To the best of our knowledge, this is the first study showing that the tumor suppressor, PML, regulates IP-10 chemokine transcription and lymphocyte trafficking in gastric cancer, by modulating the activity of the IFN-γ/STAT-1 signaling pathway.

IP-10, a lymphocyte-targeting CXC chemokine induced by type I and II IFNs, plays an important role in lymphocyte recruitment under several inflammatory conditions [41]. IP-10 is secreted by tumor-infiltrating immune cells, and also by tumoral and parenchyma cells in an interferon- and cytokine-enriched microenvironment. TAFs from lung cancer patients constitutively produce and secrete IP-10 [27]. Hepatocytes infected with the hepatitis C virus produce IP-10, and induce migration of activated T-cells to the liver parenchyma, indicating that IP-10 levels are correlated with histological severity and inflammation [42]. In our system, IP-10 secretion was observed in gastric cancer SNU-638 cells, NIH3T3 fibroblasts, and MEFs, in response to IFN-γ, and secretion was enhanced in the absence of PML protein expression. Since we found that there were no appreciable differences in the level of STAT-1 expression and phosphorylation in the absence or presence of PML upon IFN-γ (Figures 3 and 7), increased IFN-γ-induced IP-10 transcription in the absence of PML was caused mainly by enhanced STAT-1 DNA binding to IP-10 promoter. We suggest that PML-mediated inhibition of STAT-1 DNA binding leads to suppression of STAT-1-dependent IP-10 expression, in accord with our previous findings [17]. In the present study, we identify a potential regulatory region for PML in the IP-10 promoter, which is a putative STAT-1-binding site. Further, we show that PML knockdown increases IFN-γ-induced STAT-1 DNA binding to the IP-10 promoter, and enhances IP-10 levels in the absence of PML.

Lymphocytes, neutrophils, macrophages, and mast cells are the major components of most tumor infiltrates. Tumor-infiltrating lymphocytes have been identified in a variety of solid cancer tissues. However, the specific functions of such cells within tumors, and the precise molecular mechanisms of lymphocyte recruitment into tumors, remain controversial. The status and functional relevance of CD8^+^ T-cell infiltration into tumors, and the association of such cells with patient prognosis, are also unclear. In renal cell carcinoma, infiltration of CD8^+^ T-cells is associated with favorable prognosis [43]. Conversely, such infiltration is linked to unfavorable prognosis in nonsmall cell lung cancer patients [33]. We here show that PML protein expression in tumor cells is inversely correlated with the extent of infiltration of CD8^+^ T-cells into tumor tissues. When the function of PML in lymphatic invasion is considered together with the association between PML expression and unfavorable prognosis [2], it is apparent that any relationship between clinical features and the extent of CD8^+^ T-cell infiltration in gastric cancer patients requires further elucidation.

Nuclei of normal gastric mucosal glands, stromal tissues, and lymphoid cells were diffusely immunopositive (moderate-to-strong) for PML protein expression, whereas PML levels were reduced or abolished in tumor cells (Figure 1). These findings support the idea that cancer cells have specific degradation mechanisms for PML. We would propose that post-translational proteasome-dependent degradation explains the loss of PML protein. In other words, degradation at the transcriptional level is not operative [2], [44]. PML protein expression is reduced or abolished in many different cancers, including prostate, breast, CNS, colon, lung, and gastric cancer [2], [3]. Consistent with these results, we observed reduced or negligible expression of PML in advanced gastric cancer tissues. Moreover, our results suggest that cancer cells expressing different levels of PML protein vary in their capacity to attract and retain lymphocytes in tumor areas, thus providing further evidence that such cells actively participate in the establishment of inflammatory tumor microenvironments, *via* PML-mediated mechanisms.

NF-κB and STAT-3 signaling are proposed to serve as major regulatory systems linking inflammation to cancer [45]. IL-6, an NF-κB-dependent inflammatory growth factor, is essential for development of inflammation-associated carcinogenesis [46]. Recent circumstantial evidence has further strengthened the idea that the IL-6/gp130/STAT-3 signaling axis serves as both an autocrine and paracrine amplification loop in lung adenocarcinoma, multiple myeloma, Ras-transformed cancer cells, and a colitis-associated cancer model [47], [48], [49], [50]. PML has been shown to suppress IL-6-induced STAT-3 activity [51], to promote apoptosis *via* a TNF-mediated process, and to sensitize cells to apoptosis by inhibiting the NF-κB-mediated survival pathway [52]. However, it remains to be established whether loss of the tumor suppressor PML contributes to changes in the host immune response or the tumor microenvironment, perhaps resulting in either immunosurveillance or immune escape, by modulation of transcription factors. Thus, it would be interesting to compare the effects of loss of PML protein expression on NF-κB/STAT-3 and STAT-1 signaling, and to determine whether these transcription factors function in a coordinated manner to regulate the expression of a subset of chemokines contributing to the establishment of inflammatory tumor microenvironments.

## Materials and Methods

### Cells

Primary and immortalized *Pml* ^+/+^ and *Pml* ^−/−^ MEFs were established and maintained as previously described [53]. The SNU-638 human gastric carconoma cell line, Jurkat human T-cell lymphoblast-like cell line, and EL-4 mouse T-lymphoma cell lines were maintained as previously described [2], [54].

### Gastric carcinoma tissues

A total of 49 gastric carcinoma paraffin blocks were obtained from 49 patients (29 males; 20 females; median age 64 years; range 35–84) who had undergone total or subtotal gastrectomy for stage IV advanced gastric carcinomas at Mokdong Hospital, Ewha Womans University School of Medicine, Seoul, Korea from 2001 to 2007.

### Reagents and antibodies

Recombinant murine and human IFN-γ (mIFN-γ and hIFN-γ) and mIP-10 and hIP-10 were purchased from ProSpec (Rehovot, Israel). Antibodies to PML (mouse monoclonal) and STAT-1 were purchased from Upstate Biotechnology, Inc. (Lake Placid, NY, USA) and neutralizing antibody to IP-10 was purchased from R&D Systems (Minneapolis, MN, USA). Antibody to p-Y701-STAT-1 was purchased from Cell Signaling Technology (Beverly, MA). Antibodies to histone, Lamin B and PML (rabbit polyclonal) were purchased from Santa Cruz Biotechnology (Santa Cruz, CA, USA), and anti-tubulin antibody was purchased from Sigma-Aldrich, Co. (St. Louis, MO, USA).

### Immunohistochemistry, cell counts, and measurement of immunoreactive areas

Human gastric cancer tissues were processed for immunohistochemical analysis as described previously [2] with minor modifications. Primary antibodies for PML (1∶200; Santa Cruz Biotechnology), CD8 (1∶50; Thermo Fisher Scientific, Fremont, CA, USA), and IP-10 (1∶50; R&D Systems) were utilized. All slides were counterstained with Mayer's hematoxylin. Positive controls were obtained using palatine tonsils for PML protein and CD8, and nasopharyngeal undifferentiated carcinomas for IP-10. Negative controls were obtained by omitting primary antibodies from all slides. Immunopositivity for PML protein was categorized as diffuse positivity (nuclear immunoreactivity in ≥50% of tumor cells), focal positivity (in ≥10% but <50%), or complete loss (in <10% of tumor cells). The numbers of CD8 immunopositive cells in one high power field (HPF, x40) of 0.25 mm^2^ for five randomly selected tumor infiltrative borders were counted for each specimen, followed by calculation of the mean number and SD. Immunopositivity for IP-10 was defined as any nuclear or cytoplasmic positive expression in tumor cells. For determining the expressed level of each slide, digital images were obtained using a microscope-mounted digital camera. Using ImageJ software (http://rsb.info.nih.gov/ij), each image was converted to 8-bit gray scale and area densities were measured from the pixels in the region of interest; only pixels exceeding a certain level (>50 intensity value) were included to eliminate background error.

### siRNA transfection

NIH3T3 cells were transiently transfected using Mirus transfection reagent (Mirus Bio LLC, Madison, WI, USA) or Lipofectamine™ RNAiMAX (Invitrogen, Carlsbad, CA, USA) with either 300 nmol of mPML siRNA or negative control siRNA (Samchully Pharm, Co. Ltd., Seoul, Korea), according to the manufacturer's instructions. The human gastric carcinoma cell line SNU-638 was also transfected using Mirus reagent or Lipofectamine™ RNAiMAX with either 100 nmol of hPML siRNA (Santa Cruz Biotechnology) or negative control siRNA. Cells were allowed to recover for 48 h before treatment with 10 ng/ml of IFN-γ for various times, and then analyzed by immunoblotting and/or RT-PCR.

### Immunoblotting

Cells were plated, incubated with IFN-γ (10 ng/ml) for 0–24 h, and then lysed with lysis buffer [17]. Twenty µg sample of total protein or 5 µg of nuclear protein was subjected to SDS/PAGE, and blots were probed with PML, histone or tubulin antibodies. After secondary antibody incubation, blots were developed using ECL chemiluminescence system (Amersham, Buckinghamshire, UK).

### Transwell migration assay

*In vitro* migration assays were performed using Costar Transwell Inserts with a 5-µm pore size in 24-well plates (Corning Costar, Cambridge, MA, USA). SNU-638 cells were stimulated with IFN-γ (10 ng/ml) for 0–8 h, and 600 µl of the supernatants were placed in the lower chambers of transwell migration units. The upper chambers received 5×10^5^ Jurkat or EL-4 cells in a volume of 100 µl and the transwell migration units were incubated at 37°C for 2 h. The numbers of migrating cells obtained from the lower chambers were analyzed by fluorescent count-bead mediated counting on a flow cytometer (BD Biosciences, San Jose, CA, USA). For neutralization, the supernatants were pretreated with an anti-IP-10 neutralizing antibody or control non-specific IgG for 1 h, then placed in the lower chambers, and migrating cells harvested from the lower chamber were similarly determined.

### Enzyme-linked immunosorbent assay (ELISA)

Primary *Pml^+/+^* and *Pml* ^−/−^ MEFs (5×10^5^ cells/well) were seeded and treated with IFN-γ (10 ng/ml) for 12 h, then the concentration of IP-10 in supernatants was determined using a mouse IP-10 ELISA Kit (R&D Systems), according to the manufacturer's instructions. SNU-638 cells (2.4×10^5^ cells/well) were transiently transfected with either negative control siRNA or PML siRNA for 48 h, then treated with IFN-γ for 8 h. IP-10 protein levels in cell culture supernatants were measured using a human IP-10 BD optEIA ™ (BD Biosciences) and normalized by the cell protein concentration.

### RNA isolation, RT-PCR, and RPA

Total cellular RNA was isolated from confluent monolayers of cells using easy-BLUE™ (iNtRON Biotechnology, Seongnam, Korea) according to the manufacturer's protocol. cDNA was synthesized from 5 µg of total RNA with reverse transcription and PCR was performed using 3 µl cDNA with a thermal cycler (Bio-Rad Laboratories, Hercules, CA, USA). The primer sequences were as follows: mouse IP-10, forward 5′-GATGGCTAGTCCTAATTGCCCTTGG-3′, reverse 5′-CTGAGTATCTTGATAACCCCTTGGG-3′; β-actin, forward 5′-GTCACCAACTGGGACGACA-3′, reverse 5′-TGGCCATCTCTTGCTCGAA-3′. For RPA, the mCK-5 multi-riboprobe was purchased from Pharmingen (BD Biosciences) and prepared following the manufacturer's guidelines. Total RNA (15 µg) was hybridized with the riboprobe as described previously [17]. Quantification of the protected RNA fragments was performed by scanning with a PhosphorImager (Molecular Dynamics, Sunnyvale, CA, USA). Values for mRNA expression were normalized to GAPDH mRNA levels for each experimental condition.

### Plasmids, transient transfection, and reporter assays

The IP-10-luc luciferase reporter plasmids containing 330 bp of the murine IP-10 promoter were generated by PCR [55]. For overexpression of PML protein, the pCMV-Tag2B-PML-IV expression construct was used [53]. For reporter assays, 0.4 µg of the IP-10-luc construct was transiently transfected into 0.5×10^5^ NIH3T3 cells in 12-well plates, with 0.1 µg of pCMV-β-galactosidase vector to normalize for transfection efficiency as described previously [17]. The PML IV expression vector (1 µg) was co-transfected and differences in the amount of DNA adjusted with empty vector. Cells recovered for 24 h before treatment IFN-γ (10 ng/ml) for 24 h, then luciferase activity was measured. The luciferase activity of each sample was normalized to β-galactosidase activity to yield relative luciferase activity (RLA).

### Nuclear extracts and electrophoretic mobility shift assays (EMSA)

EMSA was performed with 10 µg of nuclear extracts as previously described [17]. Nuclear extracts from NIH3T3 cells treated with IFN-γ for 30 min were incubated with either the IP-10 sequence (5′-TCATCTGATTTCTCAAACAGCTCACG-3′) or consensus STAT-1 sequence (SC-2573, Santa Cruz Biotechnology) which was end-labeled with [^32^P]ATP for 30 min. For competition experiments, a 100-molar excess of unlabeled oligonucleotide was added to the nuclear extracts for 30 min before addition of the labeled probe. Bound and free DNA were then resolved by electrophoresis through a 6% polyacrylamide gel and exposed for autoradiography.

### Statistical analysis

Statistical analyses were performed using the Student's *t* test to compare between sample groups, and ANOVA was used to determine differences among multiple groups. Statistical significance of the data was set at *P*<0.05.

### Ethics statement

This study protocol was approved by the Institutional Review Board (IRB) of the Ewha Womans University Mokdong Hospital (IRB protocol number: ECT11-20-08). Informed consent had been obtained from each patient prior to surgery and was waived in this study by IRB due to the retrospective nature.
